# Mesenteric fat thickness in a group of HIV patients on HAART

**DOI:** 10.1186/1758-2652-13-S4-P72

**Published:** 2010-11-08

**Authors:** SS Lee, E Liu, KW To

**Affiliations:** 1 The Chinese University of Hong Kong, Department of Microbiology, Hong Kong, Hong Kong; 2 Prince of Wales Hospital, Department of Radiology, Hong Kong, Hong Kong; 3 Prince of Wales Hospital, Department of Medicine and Therapeutics, Hong Kong, Hong Kong

## Background

Metabolic complications of HAART are well known. Yet the structural effects are rarely described. We report the baseline carotid artery intima-media thickness (IMT), mesenteric and subcutaneous fat in a pilot study for investigating their changes following treatment with lipid lowering agents in the presence of HAART.

## Methods

All subjects were stable HIV positive patients receiving HAART. All have been attending a specialist clinic for monitoring HIV-related metabolic complications. Mesenteric, subcutaneous fat and IMT were measured by ultrasound operated by a specialist ultrasonographer. Fasting lipid was measured on the same day of ultrasound measurement. The relationships between variables were analyzed by non parametric test and univariate analysis of variance (ANOVA) where appropriate.

## Results

A total of 27 patients were recruited in the period between June 2008 and June 2009. They have been on HAART for at least 2 years. Majority was male, while. NRTI+PI, NRTI+NNRTI, NRTI+NNRTI+PI were prescribed in 16, 9 and 2 patients respectively. The triple agent group was not included in subsequent analysis because of the small sample size. About 1/5 of patients were on anti-lipid agents. The mean cholesterol, triglyceride, IMT, mesenteric and subcutaneous fat of the NRTI+PI group were 5.4+1.1 mmol/l, 3.59+2.38 mmol/l, 0.069+0.018 mm, 0.58+0.21 cm and 1.24+0.82 cm respectively. The values of NRTI+NNRTI group were 4.88+1.3 mmol/l, 3.21+2.35 mmol/l, 0.069+0.017 mm, 0.97+0.27 mm and 1.94+1.28 mm respectively. Only the mesenteric fat showed significant difference between the 2 groups, p<0.01. There was no significant relationship between lipid and IMT.

## Discussion and conclusions

Metabolic complications are commonly seen in HIV patients receiving HARRT. It is known that hyperlipidemia, increased IMT and mesenteric fat are related. However, there are little data on the effects of HAART per se on these parameters. Protease inhibitor is known to affect lipid by a class effect. Our results showed that patients receiving NRTI+NNRTI had a higher mesenteric fat than those on NRTI+PI. However, we failed to show any relationship between other parameters probably due to small sample size. Larger study would be needed to confirm our finding and whether mesenteric fat is a more sensitive marker to reflect metabolic derangement.

